# Bovine adapted transmissible mink encephalopathy is similar to L-BSE after passage through sheep with the VRQ/VRQ genotype but not VRQ/ARQ

**DOI:** 10.1186/s12917-020-02611-0

**Published:** 2020-10-08

**Authors:** Eric Cassmann, Sarah Jo Moore, Robyn Kokemuller, Anne Balkema-Buschmann, Martin Groschup, Eric Nicholson, Justin Greenlee

**Affiliations:** 1 Virus and Prion Research Unit, National Animal Disease Center, Agricultural Research Service, United States Department of Agriculture, 1920 Dayton Avenue, P.O. Box 70, Ames, IA 50010 USA; 2 Oak Ridge Institute for Science and Education (ORISE) through an interagency agreement between the U.S. Department of Energy (DOE) and the U.S. Department of Agriculture (USDA). ORISE is managed by ORAU under DOE contract number DE-SC0014664, Oak Ridge, USA; 3 Institute of Novel and Emerging Infectious Diseases, Friedrich-Loeffler-Institut, Federal Research Institute for Animal Health, —Isle of Riems, Greifswald, Germany

**Keywords:** TME, Transmissible mink encephalopathy, L-BSE, Prion diseases, Prion, *PRNP*, PrPSc, Sheep, TSE, Transmissible spongiform encephalopathies, C-BSE

## Abstract

**Background:**

Transmissible mink encephalopathy (TME) is a fatal neurologic disease of farmed mink. Evidence indicates that TME and L-BSE are similar and may be linked in some outbreaks of TME. We previously transmitted bovine adapted TME (bTME) to sheep. The present study compared ovine passaged bTME (o-bTME) to C-BSE and L-BSE in transgenic mice expressing wild type bovine prion protein (TgBovXV). To directly compare the transmission efficiency of all prion strains in this study, we considered the attack rates and mean incubation periods. Additional methods for strain comparison were utilized including lesion profiles, fibril stability, and western blotting.

**Results:**

Sheep donor genotype elicited variable disease phenotypes in bovinized mice. Inoculum derived from a sheep with the VRQ/VRQ genotype (o-bTME_VV_) resulted in an attack rate, incubation period, western blot profile, and neuropathology most similar to bTME and L-BSE. Conversely, donor material from a sheep with the VRQ/ARQ genotype (o-bTME_AV_) elicited a phenotype distinct from o-bTME_VV_, bTME and L-BSE. The TSE with the highest transmission efficiency in bovinized mice was L-BSE. The tendency to efficiently transmit to TgBovXV mice decreased in the order bTME, C-BSE, o-bTME_VV_, and o-bTME_AV_. The transmission efficiency of L-BSE was approximately 1.3 times higher than o-bTME_VV_ and 3.2 times higher than o-bTME_AV_.

**Conclusions:**

Our findings provide insight on how sheep host genotype modulates strain genesis and influences interspecies transmission characteristics. Given that the transmission efficiencies of L-BSE and bTME are higher than C-BSE, coupled with previous reports of L-BSE transmission to mice expressing the human prion protein, continued monitoring for atypical BSE is advisable in order to prevent occurrences of interspecies transmission that may affect humans or other species.

## Background

Misfolded prion proteins cause fatal neurodegenerative diseases known collectively as transmissible spongiform encephalopathies (TSEs) [1]. There are numerous TSEs affecting different species including Creutzfeldt-Jakob disease (CJD) in humans [2], bovine spongiform encephalopathy (BSE) [3], scrapie in sheep [4], and transmissible mink encephalopathy (TME) [5].

At least two TSEs of livestock species are known to be transmitted via oral consumption of contaminated feedstuffs. Classical BSE (C-BSE) in cattle is the archetypal foodborne TSE [6], and consumption of C-BSE infected cattle by humans is the most likely cause of variant CJD [7, 8] and feline spongiform encephalopathy [7, 9]. Transmissible mink encephalopathy (TME) is another foodborne TSE. Scrapie has been proposed as the origin of TME, but the exact etiology of TME is unknown [10, 11]. Based on epidemiologic and experimental investigations that occurred following a 1985 outbreak of TME in Wisconsin, Marsh and colleagues wrote in a 1991 publication that their “results suggest the presence of a previously unrecognized scrapie-like infection in cattle in the United States” [11]. Subsequent work by Baron et al. reported phenotypic similarities between L-BSE and bovine passaged TME derived from the Wisconsin outbreak [12]. We previously reported that sheep are susceptible to bovine adapted TME (bTME) after intracranial inoculation [13], and the resulting disease phenotype is similar to L-BSE in sheep with PrP^Sc^ not detected in lymphoid tissues [14, 15]. These similarities prompted an investigation to compare ovine passaged bovine TME (o-bTME) with other TSEs from cattle, C-BSE and L-BSE.

Interspecies transmission experiments are useful for investigating the origins of prion diseases and identifying possible host ranges of a prion agent. Successful interspecies transmission can result in observable alterations to the biological properties of prion strains [16–18]. Furthermore, alterations to the host range and increased transmissibility have been reported after interspecies transmission of TSEs through sheep. For example, passage of L-BSE in sheep enables transmission to wild-type mice that were not susceptible to the original bovine L-BSE isolate [19]. In another example, passage of C-BSE in sheep increases the transmission efficiency to bovinized mice compared to the original cattle C-BSE isolate [20].

Strain characteristics of TSEs can be differentiated based on a range and combination of disease features in rodents including attack rate, incubation period, fibril stability, PrP^Sc^ distribution, biochemical analysis (western blot), and vacuolar lesion distribution and severity [9, 21–26]. We investigated the effect of ovine passage of bTME in sheep of different genotypes on the disease phenotype in transgenic mice expressing the bovine prion protein. We compared these results to the disease phenotype of mice inoculated with L-BSE, C-BSE, and bTME. The results demonstrate that sheep genotype modulates the disease phenotype. Ovine passaged bTME from VRQ/VRQ genotype sheep appears more like bTME and L-BSE; whereas, inoculum from VRQ/ARQ genotype sheep is distinct from bTME and L-BSE. These findings support the model of TSE strain modulation subsequent to interspecies transmission.

## Results

### Mouse attack rates, incubation periods, and EIA

**Fig. 1:**
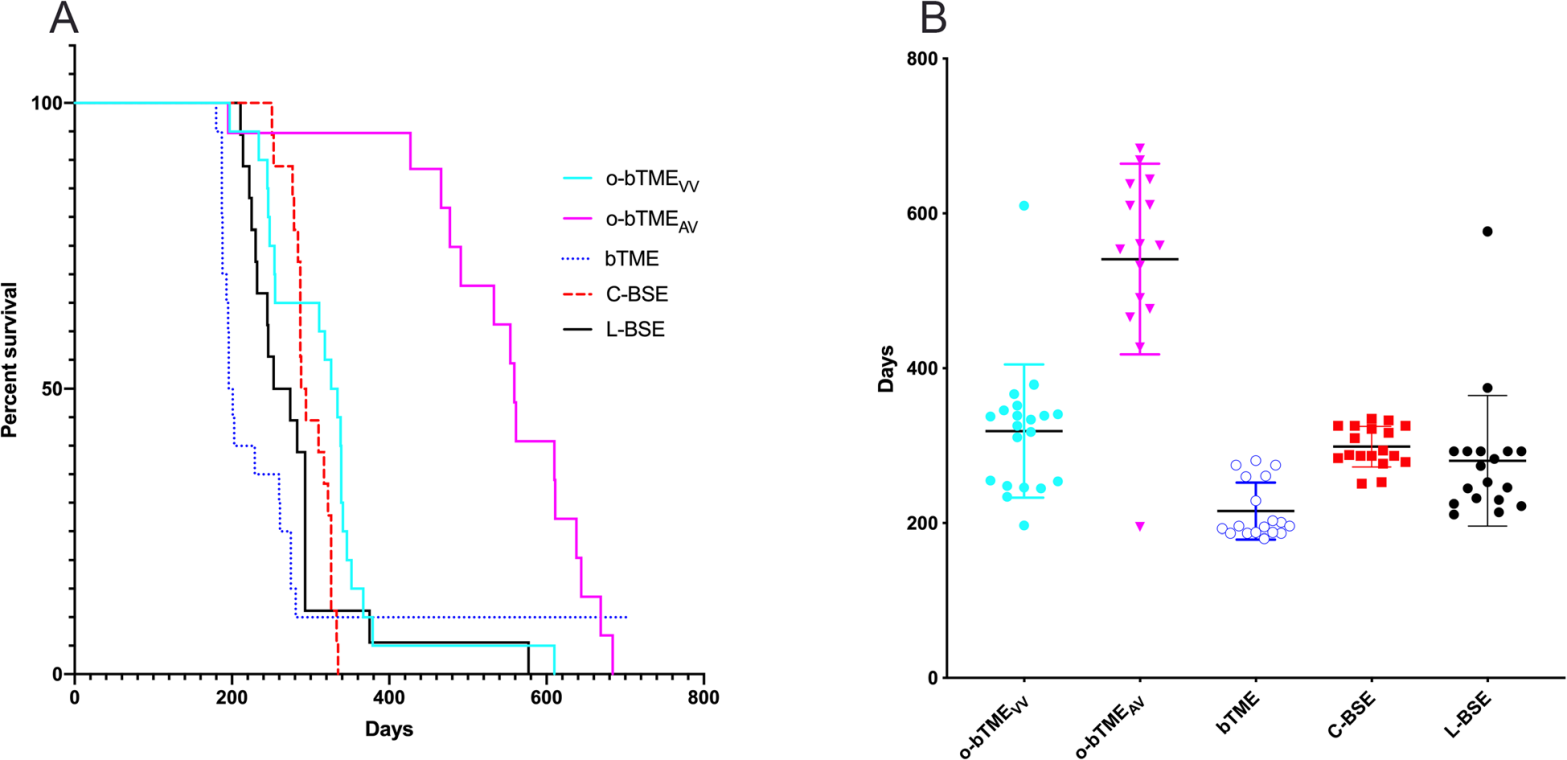
Survival and incubation. **a** Survival analysis of TgBovXV mice intracranially inoculated with one of five TSE isolates. **b** The survival times of EIA positive mice (incubation period) were similar between TME_VV_, L-BSE, and C-BSE. The center bar represents the mean and error bars represent SD

To analyze transmission efficiency and compare TSE strains, mice overexpressing bovine prion protein (TgBovXV) were inoculated with C-BSE, L-BSE, bTME, or o-bTME. The o-bTME group included two sheep genotypes: VRQ/VRQ (o-bTME_VV_) and VRQ/ARQ (o-bTME_AV_). Preliminary analysis of the experiment was performed by examining attack rates and incubation times of deceased or euthanized mice. Mice in every group developed prion disease based on clinical signs, enzyme immunoassay (EIA) results, and spongiform change. TgBovXV mice inoculated with o-bTME_VV_, o-bTME_AV_, bTME, L-BSE, and C-BSE had attack rates of 95% (20/21), 79% (15/19), 90% (18/20), 100% (18/18), and 100% (18/18), respectively. A Kaplan-Meier survival curve illustrates the longer survival time of the mice inoculated with o-bTME_AV_ (Fig. 1a). The respective mean incubation periods were 319, 541, 216, 281, and 299 days for o-bTME_VV_, o-bTME_AV_, bTME, L-BSE, and C-BSE (Fig. 1b), and the average EIA optical densities were 3.983, 3.076, 3.988, 4.0, and 4.0.

### Transmission efficiency

**Fig. 2:**
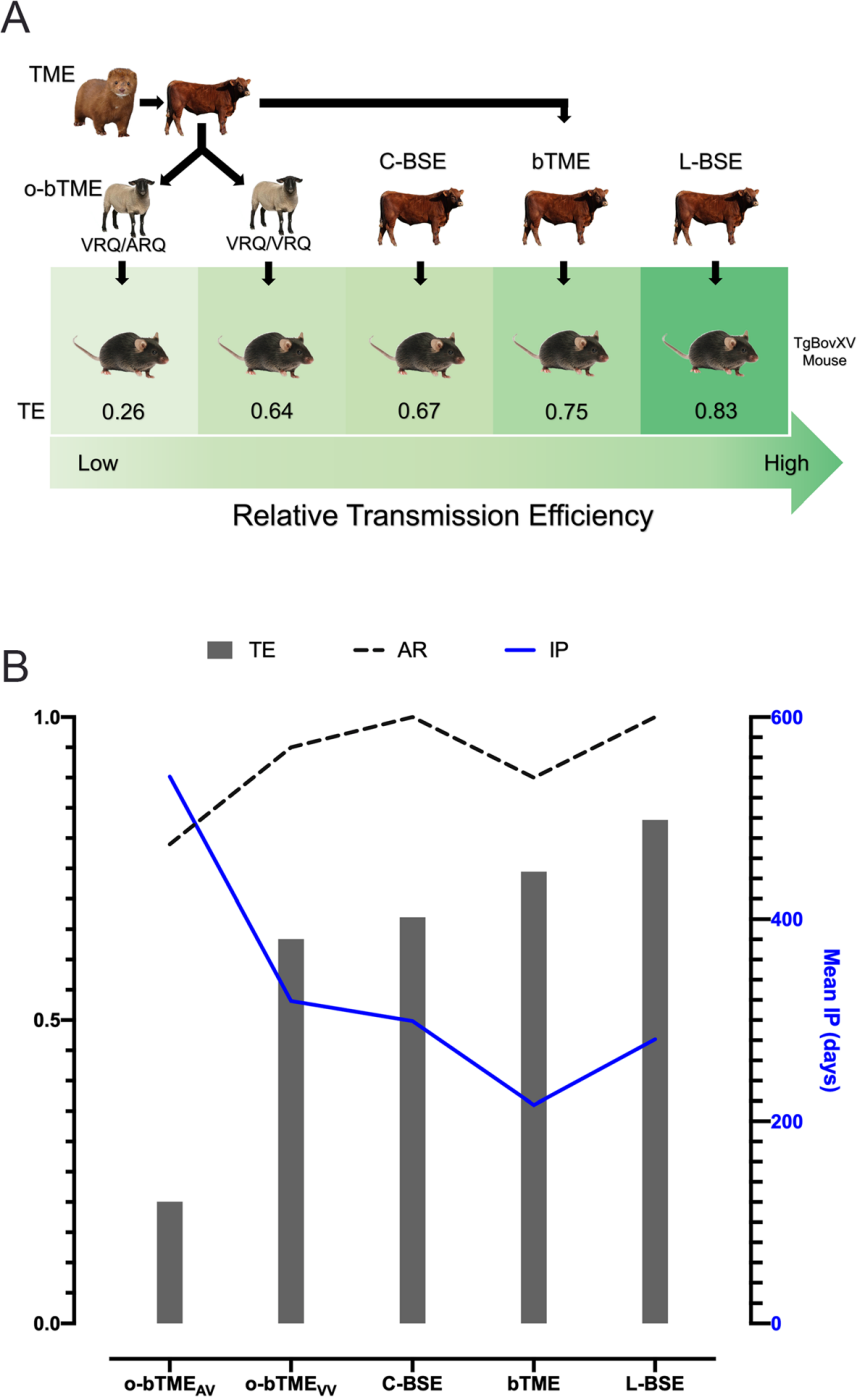
Transmission efficiency. **a** The image illustrates the sequence of interspecies transmission events of TME isolates. The relative transmission efficiencies (TE) of each isolate in TgBovXV mice is denoted by a number between 0 and 1. A value close to 1 indicates highly effective transmission. The TE ratio is calculated using attack rate (AR) and incubation period (IP). A maximum TE of 1 indicates highly efficacious transmission. Sheep genotype modulates the TE of TME to bovinized mice. **b** The relationship of transmission efficiency (TE) with attack rate (AR) and incubation period (IP) is shown. The left y-axis designates the calculated ratios for TE and AR. The right y-axis (blue) indicates the mean incubation period (IP) in days for each isolate

In order to compare the transmission efficiencies (TE) of TME isolates after multiple interspecies passages with C-BSE and L-BSE, we used an algorithm that incorporates two parameters derived from bioassays in transgenic bovinized mice (TgBovXV): attack rate (AR) and incubation period (IP) [27]. The values for attack rates and incubation periods ranged from 79 to 100% and 216–581 days, respectively. L-BSE had the highest TE in TgBovXV mice followed by bTME and then C-BSE (Fig. 2). The least efficient transmission was observed in the o-bTME_AV_ group due to a combination of a prolonged incubation period and a lower attack rate.

Ovine passaged bTME isolates were transmissible to TgBovXV mice on first passage, and the TE was influenced by the donor sheep genotype. Bovine adapted TME passaged in sheep with the VRQ/VRQ genotype had 2.5 times greater TE (TE 0.64) than sheep with the VRQ/ARQ genotype (TE 0.26).

### Neuropathology

**Fig. 3:**
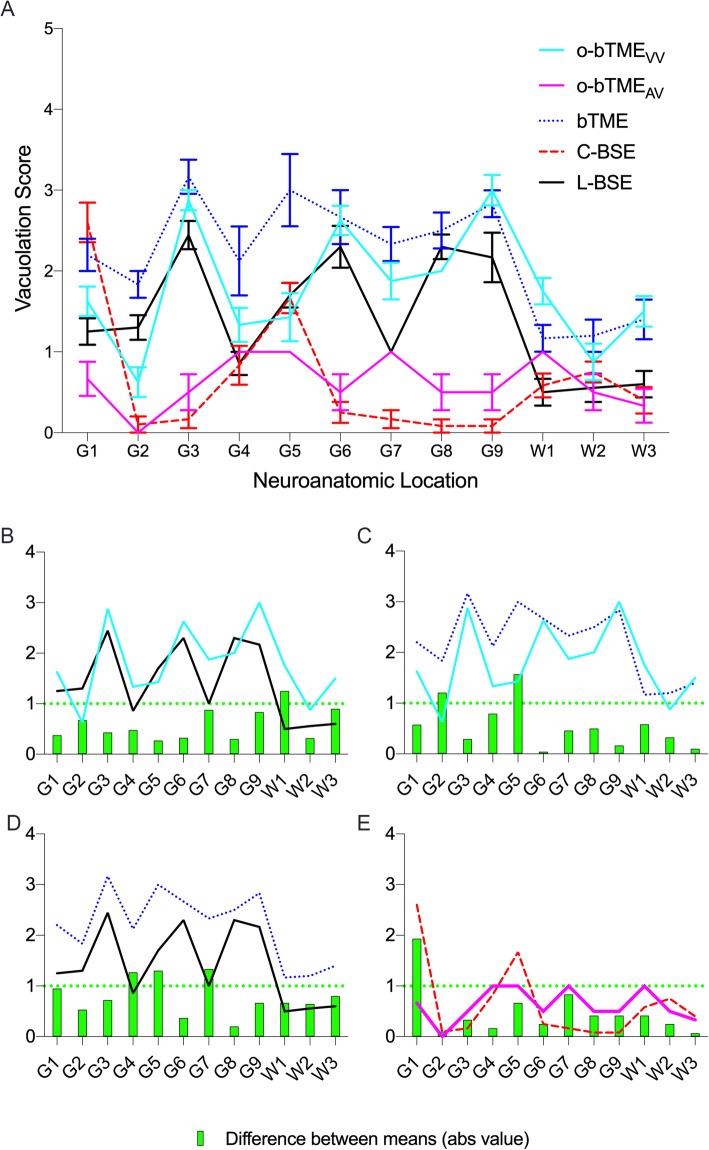
Vacuolation profiles of TgBovXV mice intracranially inoculated with various TSE strains. **a** The degree of vacuolation was plotted (mean ± SEM) against predefined neuroanatomic locations to generate the lesion profile. The pattern of the profiles is most similar between o-bTME_VV_ (solid cyan line), L-BSE (solid black line), and bTME (dotted blue line) with some minor variations in the degree of vacuolation. C-BSE (red dashed line) and o-bTME_AV_ (solid pink line) have distinct lesion patterns compared to bTME, o-bTME_VV_, and L-BSE. Vacuolation profiles are displayed with the absolute values of the difference between the plotted means (green bar) of (**b**) o-bTME_VV_ vs. L-BSE, (**c**) o-bTME_VV_ vs. bTME, (**d**) L-BSE vs. bTME, and (**e**) o-bTME_AV_ vs. C-BSE. A horizontal dashed-green line intersects the y-axis at 1 indicating a subjective threshold to gauge the relative intensity of vacuolation. Medulla (G1), cerebellum (G2), midbrain (G3), hypothalamus (G4), thalamus (G5), hippocampus (G6), para terminal body (G7), and cerebral cortex (G8 and G9). White matter in the cerebellar peduncle (W1), lateral tegmentum (W2), and the internal capsule (W3)

Vacuolation lesion profiling was used to compare the degree of vacuolation in ovine passaged bTME with bTME, L-BSE, and C-BSE according to previously described methods [28]. A minimum of six mice per group were used to generate lesion profiles (Fig. 3a); some mouse to mouse variation was present in each cohort (Additional file 1). The overall patterns of lesion severity were broadly similar between o-bTME_VV_, bTME, and L-BSE, and they were distinct from both o-bTME_AV_ and C-BSE. The average lesion scores of L-BSE and o-bTME_VV_ varied by a maximum of 0.875 to 1.2 across three neuroanatomical locations (Fig. 3b). The areas with the greatest differences in mean lesion scores were the paraterminal body (G7), white matter of the cerebellar peduncle (W1), and internal capsule (W3) corresponding to differences of 0.875, 1.25, and 0.9, respectively. Only a single location, W1, varied by a score greater than 1. For o-bTME_VV_ and bTME (Fig. 3c), two locations had notable differences in vacuolation: the cerebellum (G2) and thalamus (G5). The four highest differences between the lesion scores of bTME and L-BSE ranged from 0.95 to 1.3 (Fig. 3d). The four locations with the greatest differences in vacuolation included the medulla (G1), hypothalamus (G4), thalamus (G5), and para terminal body (G7). For o-bTME samples, the distribution and severity of vacuolar change was dependent on the sheep donor genotype (VV_136_ vs. AV_136_). The pattern of o-bTME_VV_ was distinct from o-bTME_AV_. In the midbrain (G3), hippocampus (G6), and cerebral cortex at the level of the septal nuclei (G9), the difference in mean vacuolation scores was 2.4, 2.1, and 2.5, respectively. The difference was greater than 1 but less than 2 in the cerebral cortex at the level of the thalamus (G8) and the internal capsule white matter (W3). The lesion profiles of mice inoculated with o-bTME_AV_ and C-BSE had mean differences of less than 1 in 11/12 locations (Fig. 3e); in the medulla (G1), C-BSE inoculated mice had more severe vacuolation than o-bTME_AV_ (mean difference 1.9).

**Fig. 4:**
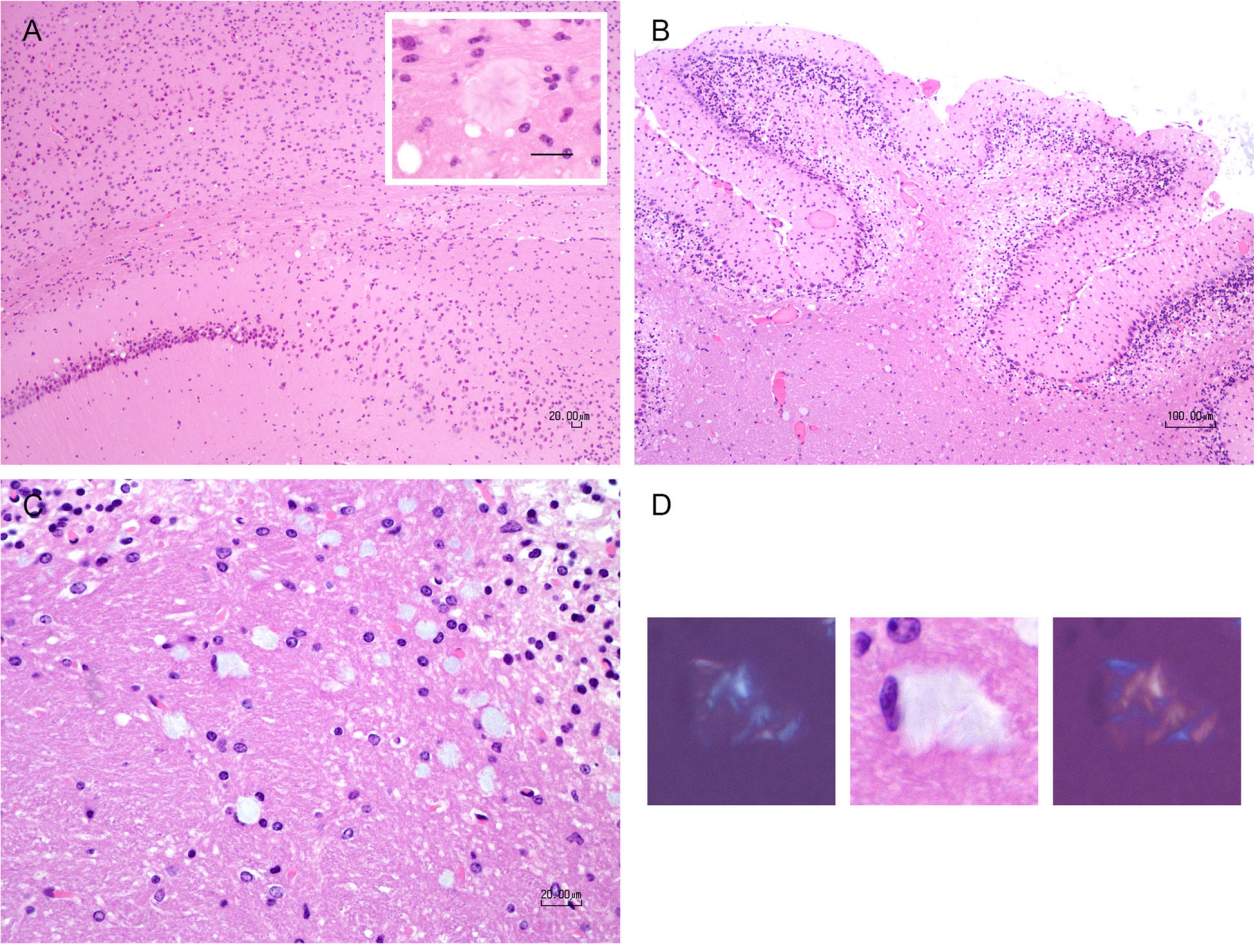
Neuropathology. **a** C-BSE. Florid plaques were visible at the level of the hippocampus. Mild spongiform change also was present in this region. Inset: magnified view of a florid plaque. Bar = 20 μm. **b** L-BSE. There was marked spongiform change present in the granule cell layer and at its interface with white matter of the cerebellum. Granule cell depletion was striking. **c** L-BSE. Magnified view of the location in image B demonstrating non-florid plaques in the white matter. **d** L-BSE. A non-florid plaque with hematoxylin and eosin staining characterized by amphophilic color and a fine, linear, crisscross pattern. Polarization of light through the non-florid plaque demonstrated birefringent dichroism

There were differences in the presence and severity of other neuropathology between groups. Mice that received C-BSE inoculum had florid plaques in the hippocampus (Fig. 4a). Those inoculated with C-BSE, L-BSE, bTME, and o-bTME had mild to prominent non-florid plaque accumulations characterized by amphophilic, thin, linear cross-hatching that was visible with routine hematoxylin and eosin staining (Fig. 4d). They were most prevalent in the white matter of the cerebellum. The non-florid plaques also exhibited birefringent dichroism observable with polarized light microscopy. Finally, a subset of TgBovXV mice inoculated with L-BSE had advanced granule cell depletion in the cerebellum.

### Immunohistochemistry for PrP^Sc^

**Fig. 5:**
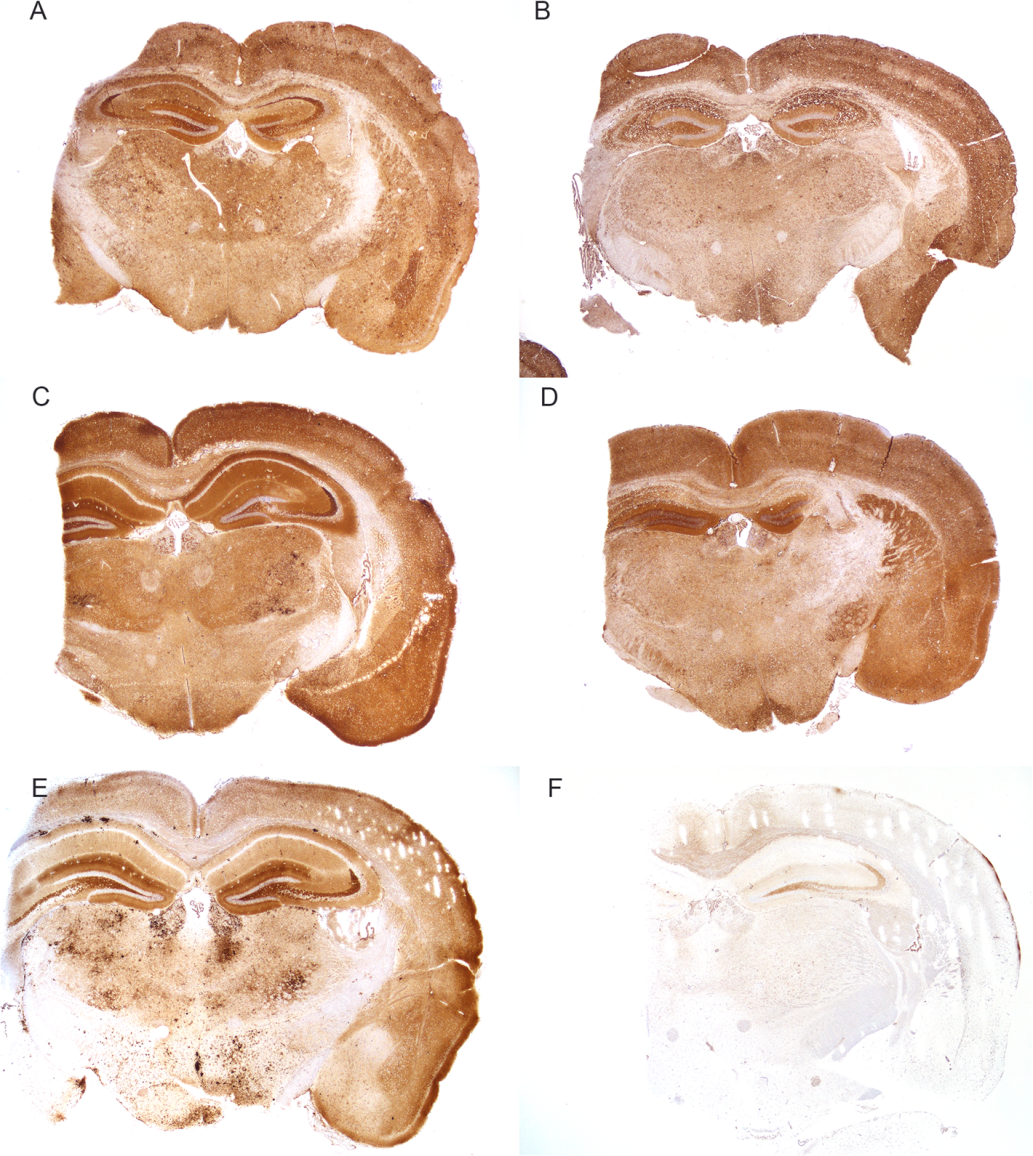
Immunohistochemistry. Patterns of PrP^Sc^ immunoreactivity in the brains of TgBovXV mice inoculated with (**a**) bTME, (**b**) L-BSE, (**c**) o-bTME_AV_, (**d**) o-bTME_VV_, and (**e**) C-BSE. There was diffuse immunoreactivity in the neuropil of the cerebral cortex, thalamus, hypothalamus, and hippocampus of bTME, L-BSE, o-bTME_AV_, and o-bTME_VV_. Granular particulate coalesces in the thalamus and cerebral cortex of b-TME, L-BSE, and o-bTME_AV_. C-BSE contains PrP^Sc^ associated with plaques in the corpus collosum, hippocampus, thalamus, and hypothalamus. **f** A non-inoculated TgBovXV mouse had mild non-specific immunoreactivity in the polymorph layer of the dentate gyrus, cerebral cortex, endothelial cells, and choroid plexus epithelium. PrP^Sc^ detected with monoclonal antibody 6C2

The pattern of PrP^Sc^ deposition was compared at the level of the thalamus for each inoculation group (Fig. 5). Observable differences were present in the cerebral cortex, hippocampus, thalamus, and hypothalamus. In C-BSE inoculated mice there were multifocal plaques of PrP^Sc^ in the corpus collosum, hippocampus, thalamus, and hypothalamus. In comparison, L-BSE, bTME, and both o-bTME strains lacked plaques; instead the thalamus and sometimes the cerebral cortex contained aggregates of granular PrP^Sc^. Coalescing granular PrP^Sc^ was more notable in the thalamus of o-bTME_AV_. Diffuse particulate was present in the neuropil of the cerebral cortex, thalamus, and hypothalamus in L-BSE, bTME, and both o-bTME strains. Each of these strains also had more immunoreactivity in CA1 of the hippocampal formation while PrP^Sc^ in C-BSE inoculated mice was most severe in the molecular layer of the dentate gyrus in the hippocampal formation. There was mild nonspecific binding in non-inoculated TgBovXV mice in the polymorph layer of the dentate gyrus in the hippocampal formation; some nonspecific binding was also observed in endothelial cells, choroid plexus epithelium, and minimally in the cerebral cortex.

### PrP^Sc^ fibril stability

**Fig. 6:**
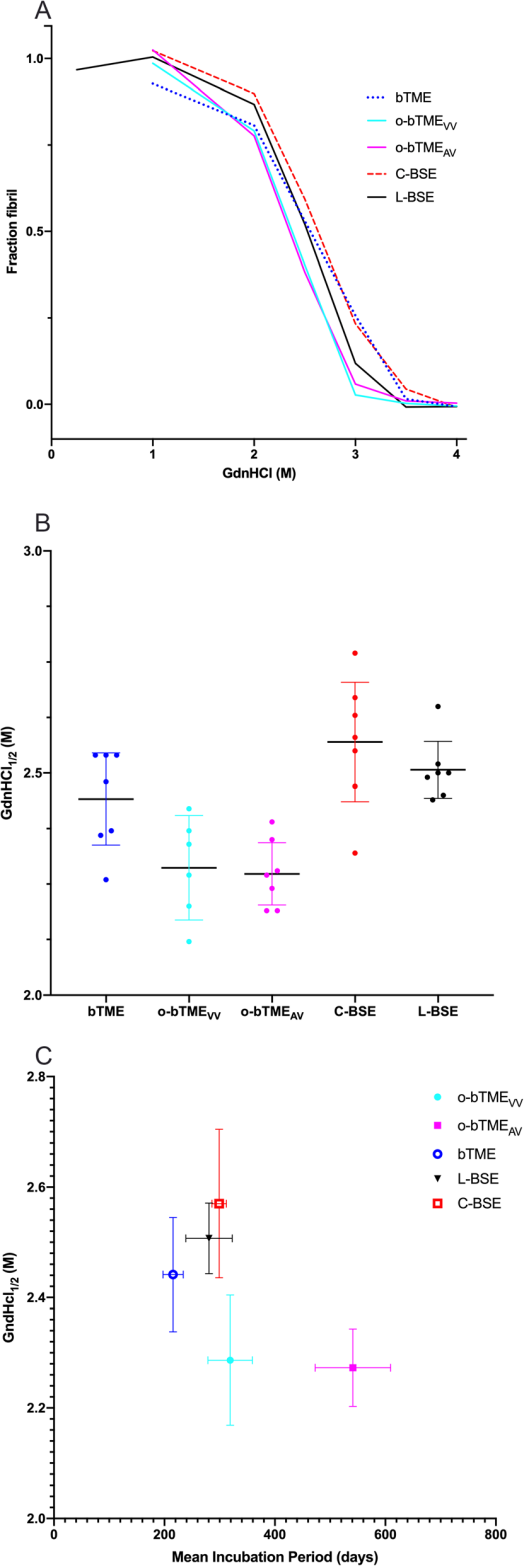
PrP^Sc^ fibril stability analyses. **a** Fibril stability curves. Representative fibril unfolding curves for the unfolding assay are shown as a smoothed trace of the fraction fibril as a function of GdnHCl concentration for bTME, o-bTME_VV_, o-bTME_AV_, C-BSE, and L-BSE. Only a single representative curve is shown here for clarity; the mean fibril midpoints shown in panel B are preferable for evaluating differences in the fibril stabilities. **b** Fibril folding midpoints. The fibril unfolding midpoints are shown for the indicated sample types (mean ± 95% CI). The midpoint is the concentration of GdnHCl resulting in a 0.5 fraction fibril remaining. Each mean was the determined from seven independent GdnHCl_1/2_ concentrations and measurements. **c** The fibril stability midpoints are plotted against the mean incubation periods for each inoculation group. Ovine passaged bTME isolates exhibited lower stabilities with longer incubation periods after first round transmission to TgBov mice. Bovine adapted TME, L-BSE, and C-BSE had higher fibril stabilities and shorter incubation periods compared to o-bTME

The fibril stability was determined by graduated digestion with guanidine hydrochloride (GdnHCl). The midpoint fibril fraction remaining was not significantly different between o-bTME_VV_ and o-bTME_AV_ (*p* = 0.9993). C-BSE and L-BSE required higher concentrations of GdnHCl to reduce the fibril fractions of PrP^Sc^ to 0.5 (Fig. 6). This translated to significant differences between the PrP^Sc^ fibril stabilities of C-BSE and L-BSE compared to both types of ovine passaged bTME (o-bTME_VV_ vs. C-BSE *p* = 0.0004; o-bTME_VV_ vs. L-BSE *p* = 0.0069; o-bTME_AV_ vs. C-BSE *p* = 0.0001; o-bTME_AV_ vs. L-BSE *p* = 0.0025). Bovine passaged TME had an intermediate fibril stability that was not significantly different from o-bTME_VV_, C-BSE, or L-BSE (*p* > 0.05); however, the confidence of a difference between bTME and o-bTME_AV_ was significant (*p* = 0.0440). The lower fibril stability of o-bTME_AV_ corresponded to a longer incubation period; whereas, C-BSE and L-BSE had shorter mean incubation periods and higher fibril stabilities.

### Western blot

**Fig. 7:**
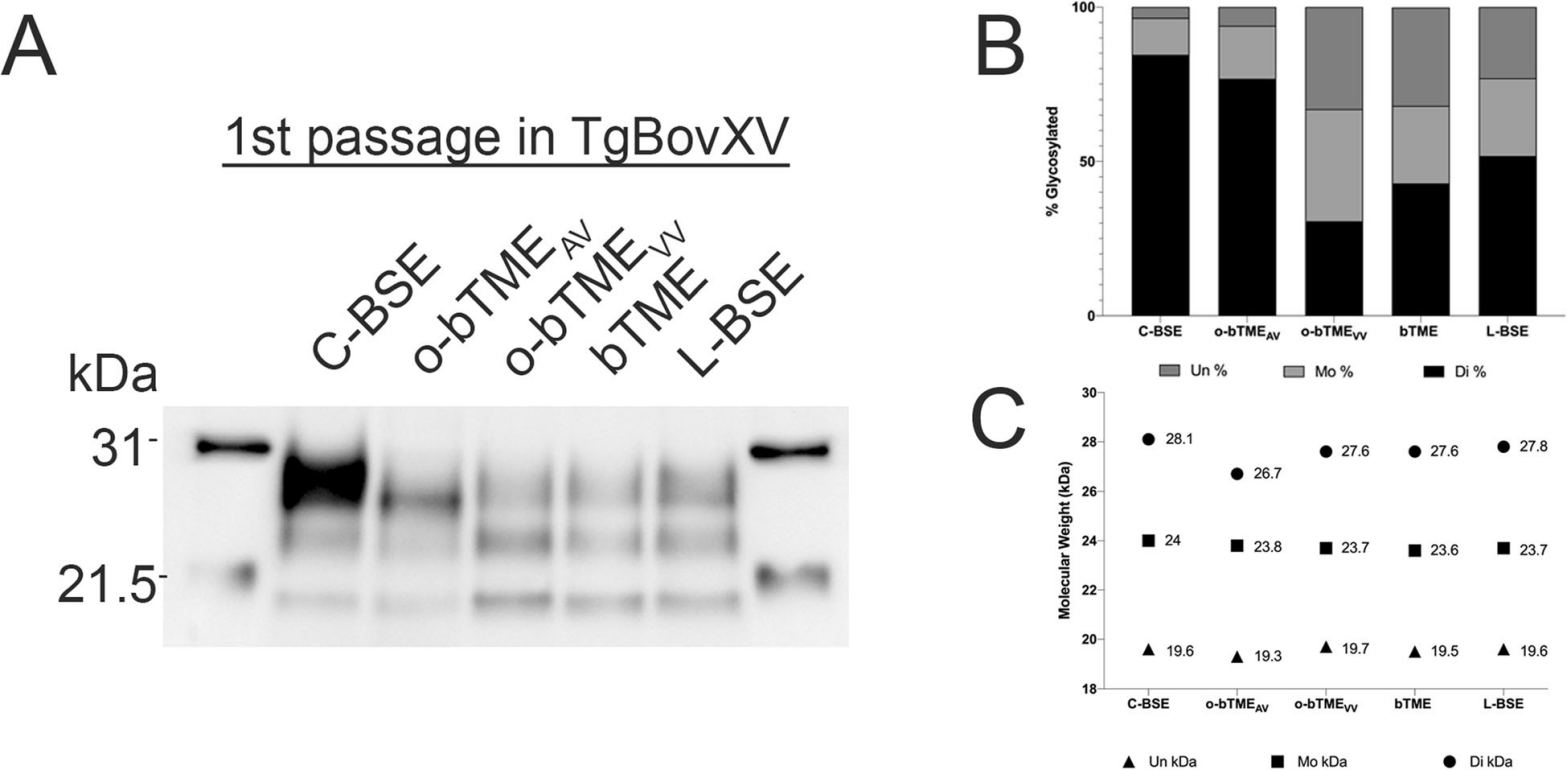
**a** Western blot was used to assess the molecular characteristics of PK-resistant PrP material in the brains of TgBovXV mice. Samples from o-bTME_VV_, bTME, and L-BSE inoculated mice were similar to each other and different from C-BSE. The antibody 6H4 anti-prion antibody was used for detection (1:10,000). **b** Glycosylation analysis of the western blot from panel A. The percentage of diglycosylated PrP^Sc^ comprised the majority of signal in the C-BSE sample. Likewise, the amount of diglycosylated PrP^Sc^ in o-bTME_AV_ was much higher compared to o-bTME_VV_, bTME, and L-BSE. **c** Molecular weight analysis of the western blot from panel A. The molecular weight of the diglycosylated band of o-bTME_AV_ was less than other isolates

In order to compare the molecular profiles of each TSE inoculum group, we performed western blots. We assessed the isolates for differences in their size and glycoform ratios (Fig. 7; Additional file 2). The migration patterns were similar between all isolates except that o-bTME_AV_ had a slightly smaller diglycosylated fragment (26.7 kDa). The o-bTME_VV_, bTME, and L-BSE glycoform ratios were similar; however, the o-bTME_AV_ derived samples had more diglycosylated PrP^Sc^ than o-bTME_VV_, L-BSE, and bTME.

## Discussion

Mouse bioassays are commonly used to characterize prion strains. We inoculated transgenic mice that overexpress bovine PrP^C^ with bovine adapted TME (bTME), ovine passaged bTME, L-BSE, and C-BSE and compared the incubation period, attack rate, vacuolation lesion profile, amount of PrP^Sc^, PrP^Sc^ pattern, western blot profile, and PrP^Sc^ fibril stability for each inoculum. These data were used to evaluate strain differences and transmission efficiencies of selected TSEs. We were interested in determining the effects of multiple interspecies transmission events on strain phenotype. We found that both the TSE agent-type and the sheep donor genotype influenced the transmission efficiency. We also observed that o-bTME_VV_, bTME, and L-BSE shared phenotype characteristics, and o-bTME_AV_ was different from o-bTME_VV_, bTME, and L-BSE.

To compare the effectiveness of prion strains to replicate and cause disease in mice, several outcomes are easily measurable including the attack rate (AR) and incubation period (IP). These parameters are considered partially representative of the strain phenotype. Between L-BSE, bTME, and o-bTME_VV_, there was little disparity between ARs and IPs. For example, the AR was slightly higher in o-bTME_VV_ compared to L-BSE, but the IP was longer compared to bTME. Recently, a computative ratio called the *Transmission Efficiency* (TE) combined these parameters into a single number [27]. L-BSE had the highest TE in TgBovXV mice followed by bTME. The TE of o-bTME_VV_ was similar to C-BSE; however, o-bTME_AV_ had the lowest TE. The less efficient transmission of o-bTME compared to bTME is most likely due to the species barrier effect (sheep PrP to bovine PrP); whereas, the other isolates represent intraspecies passages of bovine prion protein to bovinized mouse prion protein. Nonetheless, the high attack rates and high EIA results for o-bTME isolates indicate the absence of a robust species barrier.

The phenomenon of altered transmissibility after passage through an intermediate species has been previously documented. For example, the passage of classical BSE through sheep results in a decreased incubation period in BoPrP-Tg110 mice [20] and increased susceptibility of human-PrP transgenic mice [29]; atypical BSE isolates adapted in sheep also have increased zoonotic ability in human-PrP transgenic mice [30]. Furthermore, transmission of TSEs to intermediate species can expand the host range to include species that were not susceptible to the original TSE [19, 31]. Additional studies would be necessary to determine the complete host range of TME after modification by interspecies transmission.

The sheep donor genotype also influences the efficacy of interspecies prion transmission. Ovine bTME isolates from VRQ/VRQ and VRQ/ARQ genotype sheep have notably different TEs in bovinized mice. The bovine adapted TME agent has a high attack rate in bovinized mice inoculated with brain homogenate from VRQ/VRQ genotype sheep; however, the transmission efficiency is reduced after passage through sheep with the VRQ/ARQ genotype. This pattern of host genotype versus disease susceptibility is similar to that observed in classical scrapie-affected sheep [21, 32–36]. The present study demonstrates that sheep donor genotype also influences the transmission efficiency of a non-scrapie TSEs to other species.

Divergent neuropathology and western blot profiles arose after passage of bovine adapted TME through different ovine host genotypes: VRQ/VRQ and VRQ/ARQ. Since other authors have reported similarities between L-BSE and bovine passaged TME from the Stetsonville, WI outbreak [12, 13], we compared the ovine passaged isolates to L-BSE in TgBovXV mice. The VRQ/VRQ donor material (o-bTME_VV_) had a similar pattern and vacuolar lesion scores to L-BSE and bTME. Both bTME and o-bTME_VV_ had similar western blot migration patterns to L-BSE. The lesion profile of o-bTME_AV_ was notably different compared to o-bTME_VV_, bTME, and L-BSE. Another difference with o-bTME_AV_ isolates was a larger fraction of diglycosylated PrP^Sc^. Even though o-bTME_AV_ and C-BSE had similar glycosylation fractions, 77 and 85%, respectively, the molecular weight of the diglycosylated band in C-BSE was greater than o-bTME_AV_. Brains from C-BSE inoculated mice also contained florid plaques that were absent from o-bTME_AV_ and other isolates.

To evaluate the transmission efficiency between species, a first-passage transmission study is compulsory. In this study, ovine passaged bTME is a first-passage interspecies transmission event. However, the effect of host adaptation on disease phenotype cannot be fully accounted for in the lesion profiles derived from first passage mice. Subsequent repeated intraspecies passages is required to sift out strain variants and select for a new variant [16, 18, 37–42]. This strain selection results in a decreased IP and stabilized neuropathology [43]. Subsequent intraspecies passages would result in increased severity of vacuolation with retention of regional distribution [18]. Therefore, a shortcoming of the present study is the use of first passage interspecies transmission mice to construct lesion profiles. The lesion profiles of C-BSE and o-bTME_AV_ had similar regional distribution, but o-bTME_AV_ generally had more severe vacuolation than C-BSE. Consequently, upon subsequent passages, it could be expected that the magnitude of vacuolation would increase further for o-bTME_AV_ leading to a greater discrepancy with C-BSE. To the contrary, vacuolation of o-bTME_VV_ is usually less compared to bTME; therefore, repeated passages in TgBovXV mice would be expected to increase the degree of vacuolation possibly making lesion profiles for o-bTME_VV_ and bTME more similar. To evaluate these hypotheses, second passage transmissions of o-bTME in TgBovXV mice are planned.

The patterns of immunoreactivity to PrP^Sc^ have been used to compare and differentiate prion strains in mice [44–46]. In this study, PrP^Sc^ distribution for C-BSE was distinct from L-BSE, bTME, and o-bTME_VV_ and o-bTME_AV_. Interestingly, the pattern of PrP^Sc^ distribution in o-bTME_VV_ and o-bTME_AV_ was similar despite these strains having largely discrepant lesion profiles. A first passage effect wouldn’t account for the difference in lesion profiles between o-bTME_VV_ and o-bTME_AV_ since both strains originated from an ovine donor. Instead, differences in the lesion profile arose after first passage while PrP^Sc^ deposition patterns remained similar. Still, there were similarities in the PrP^Sc^ distribution between L-BSE, bTME, and both ovine bTME strains.

We sought to compare the PrP^Sc^ fibril stabilities of bovine spongiform encephalopathies with host adapted bTME and non-host adapted ovine bTME. The stabilities of the non-host adapted o-bTME isolates were different than C-BSE and L-BSE; whereas, bovine adapted TME fibrils displayed intermediate stability. Previous work has demonstrated differences in the fibril stabilities between prion strains [47, 48]. In the present experiment, the midpoint of the GdnHCl fibril unfolding curve of bovine adapted TME was not significantly different from C-BSE and L-BSE. The o-bTME isolates were indistinguishable from each other. Therefore, fibril stability analysis wasn’t an all-inclusive means for strain differentiation in this experiment.

Our findings differed from previous work that demonstrated a positive correlation between incubation period and conformational stability in mice inoculated with various TSE isolates [49, 50]. Namely, shorter incubation periods in mice were associated with lower stability. Lower conformational stability is postulated to allow increased exposure of PrP^Sc^ to bind PrP^C^ resulting in more rapid propagation of PrP^Sc^ that shortens the incubation period [51]. This correlation has been similarly identified in naturally occurring TSEs [52, 53]; however, in other laboratory animal models of prion disease, different observations have been made. Short incubation hamster-adapted prion strains have higher fibril stability while strains with longer incubation periods exhibit lower fibril stability [54]. In the present work, three isolates most recently passaged in cattle exhibited higher fibril stability and shorter incubation periods compared to ovine passaged isolates. The two ovine passaged bTME isolates exhibited no significant difference in fibril stability compared to each other despite having different incubation periods. The o-bTME_VV_ isolate had an incubation period similar to L-BSE and C-BSE; however, its fibril stability was lower. Our observations could be due to species barrier effects. It is possible that subsequent passage and host adaptation could result in changes to either fibril stability or incubation time.

## Conclusions

Various factors including TSE agent, interspecies transmission, and sheep donor genotype can influence the transmission efficiency and disease phenotype in this bovine model. In this case, the western blot and lesion profiles of o-bTME_AV_ differed greatly from o-bTME_VV_, bTME, and L-BSE. The lesion profile, PrP^Sc^ patterns, and western blot similarities between L-BSE, bTME, and o-bTME_VV_ support the hypothesis of a common origin for these prion strains. Previous work has shown that the Stetsonville, WI outbreak of TME could have been precipitated by feeding mink a downer cow with atypical BSE; therefore, it very well may have originated from a cow with L-BSE. The agent of TME appears to remain stable, and it has a high transmission efficiency after a sequence of interspecies transmission events. Although C-BSE is the archetypal foodborne TSE, our findings indicate that L-BSE and bTME have greater transmission efficiencies in bovinized mice. Previous work has demonstrated that L-BSE also is more virulent than C-BSE in mice expressing the human prion protein [46, 55]. Although the documented incidence of L-BSE is low, the propensity of L-BSE and the TME agent to cross species barriers support the continued monitoring for atypical BSE.

## Methods

### Ethics statement

This study was done in accordance with the Guide for the Care and Use of Laboratory Animals (Institute of Laboratory Animal Resources, National Academy of Sciences, Washington, DC, USA) and the Guide for the Care and Use of Laboratory Animals in Research and Teaching (Federation of Animal Science Societies, Champaign, IL, USA). The protocol was approved by the Institutional Animal Care and Use Committee at the National Animal Disease Center (protocol number: ARS-2017-628).

### Inoculum sources and preparation

The inocula for this experiment were derived from experimental studies and field isolates. The agent of transmissible mink encephalopathy was previously passaged in cattle (bTME) three times [56] and passaged to sheep [13]. Two separate groups of ovine passaged bTME (o-bTME) were derived from sheep with different prion protein genotypes: VRQ/VRQ (o-bTME_VV_) and VRQ/ARQ (o-bTME_AV_). Classical bovine spongiform (C-BSE) encephalopathy and L-type BSE (L-BSE) samples were obtained from field cases in the U.S. (2003) and France (2005) [57], respectively. The final inocula were prepared as 1% (w/v) homogenates using sterile phosphate-buffered saline.

### Transgenic mice, inoculation, endpoints, and sample processing

We used a transgenic mouse model to compare the pathologic phenotypes of bTME, o-bTME, C-BSE, and L-BSE. Transgenic mice overexpressing bovine PrP^C^ (TgBovXV) were obtained from the Friedrich-Loeffler-Institut (Federal Research Institute for Animal Health, Greifswald—Isle of Riems, Germany) [58]. Experimental groups included 18–21 mice per inoculum group; a minimum sample size of 15 mice is recommended for studies with expected long incubation periods [59]. Mice were anesthetized with isoflurane and inoculated intracranially with 20 μL of a 1% w/v brain homogenate derived from clinically diseased animals previously confirmed to have a TSE by EIA, western blot, and anti-PrP^Sc^ IHC. Approval from the Institutional Animal Care and Use Committee was procured prior to conducting this experiment (protocol number ARS-2017-628).

Following inoculation, all mice were housed in a biosafety level 2 or 3 facility (3 for BSE inoculated animals) and fed a pelleted rodent ration with access to water ad libitum. They were co-housed in cages specific to their inoculum group. Mice were examined daily for potential signs of prion disease including poor hygiene/haircoat, ataxia, circling, or inability to right position. Upon discovery of clinical signs, the animals were humanely euthanized, and routine samples were collected. When death resulted from intercurrent disease, samples were also collected, but specific criteria (see *survival analysis*) were used to determine which mice were used to calculate incubation periods. At the predetermined experimental endpoint of approximately 700 days, any unaffected/asymptomatic mice were humanely euthanized. The methods of euthanasia approved and used for these experiments were inhalation of carbon dioxide gas or anesthesia with isoflurane followed by decapitation in accordance with the AVMA Guidelines for the Euthanasia of Animals and the Guide for the Care and Use of Laboratory Animals (Institute of Laboratory Animal Resources, National Academy of Sciences, Washington, DC, USA). An enzyme immunoassay (EIA) was used as a screening test for prion disease for all mice. To complement the results of diagnostic EIA, western blotting, and hematoxylin and eosin stained sections of brain confirmed the presence of spongiform encephalopathy and neuropathology consistent with a diagnosis of TSE.

For sample collection, a 2/3 longitudinal section of the brain was fixed in 10% buffered neutral formalin, and the other 1/3 was frozen for downstream EIA and guanidine hydrochloride fibril stability analyses. Only brains from recently euthanized mice were used for microscopic examination. Formalin fixed brains were transected at the levels of the frontal cortex, hippocampus, midbrain, and medulla oblongata resulting in five sections [28] that were paraffin embedded and sectioned to 4 μm thickness. Sections were placed on glass slides and stained with hematoxylin and eosin.

### Enzyme immunoassay

Enzyme immunoassay (EIA) was carried out similarly to previously described methods [13, 60] using a commercially available enzyme-linked immunoassay (HerdChek®, IDEXX Laboratories Inc., Westbrook, ME). Frozen brain samples were prepared as a 20% (w/v) tissue homogenates and treated with proteinase K. From that point, the assay was completed according to kit instructions. Cut-off numbers were determined with a negative control as per the kit instructions; values greater than the mean optical density (O.D.) of negative controls + 0.180 were considered positive.

### Survival analyses

To calculate the mean incubation period (IP) in mice that died prior to the experimental endpoint, we averaged the survival times of EIA positive mice and removed outliers beyond three standard deviations from the mean. Any mice that died preceding three standard deviations from the mean were not included in the IP calculation since their incubation time was artificially shortened due to intercurrent disease. However, the attack rates (AR) were calculated by including all EIA positive mice in the numerator. The denominator of AR was determined after censoring EIA negative mice that died earlier than three standard deviations less than the mean IP (early intercurrent disease). Analyses for all experiments were performed using Microsoft Excel (Microsoft Office, Redmond, WA) and GraphPad Prism 7 (GraphPad Software, San Diego, CA).

### Transmission efficiency calculations

In order to compare the transmission efficiencies (TE) of different TSE isolates to transgenic mice, we utilized a computational model that incorporates attack rate (AR) and incubation period (IP) [27]. Determination of the IP multiplier was performed in alignment with the method described by Nonno and colleagues [27] based on the duration of survival in TSE positive mice (incubation period). There were six possible IP multiplier categories with corresponding values of 1, 0.83, 0.67, 0.50, 0.33, and 0.17 that were assigned based on the average incubation period in days: < 200, 200–299, 300–399, 400–499, 500–599, and > 600, respectively.

### Microscopic examination and lesion profiling

Spongiform change was evaluated with hematoxylin and eosin stained sections of brain using brightfield microscopy. The severity of vacuolation was scored on a scale from 0 to 5 in predefined grey matter locations as previously described [28]. The score (magnitude of vacuolar change) was plotted against neuroanatomic location to construct a lesion profile. Grey matter areas included the medulla (G1), cerebellum (G2), midbrain (G3), hypothalamus (G4), thalamus (G5), hippocampus (G6), para terminal body (G7), and cerebral cortex (G8 and G9). White matter in the cerebellar peduncle (W1), lateral tegmentum (W2), and the internal capsule (W3) were also evaluated for spongiform change [61, 62] on a scale from 0 to 3. A minimum of six mice were scored per group based on a single observer. Mean scores were plotted with error bars representing the standard error of the mean (GraphPad Prism 7, GraphPad Software, San Diego, CA). Differences between the means greater than one were generally considered to indicate substantial differences in vacuolation.

### Immunohistochemistry for PrP^Sc^

In order to assess the patterns of PrP^Sc^ accumulation in the brains of mice inoculated with different TSE agents and strains, we performed immunohistochemistry on formalin fixed paraffin embedded brain sections. Slides were rehydrated with xylene and ethanol. Antigen retrieval was performed in TE buffer (10 mM Tris Base, 1 mM EDTA, 0.05% Tween 20, pH 9.0) held at 121 °C for 20 min in an autoclave. Slides were then treated with 10% formic acid for 10 min. The rest of the protocol was performed on a BOND-Max automated immunohistochemistry stainer (Leica Biosystems, Buffalo Grove, IL) with 3–6 washes between steps. Proteinase K was applied for 7 min (20 μg/mL in TE buffer, pH 8.0). Non-specific protein binding was blocked by incubating with Background Buster for 30 min (Innovex Biosciences, Richmond, CA). The primary antibody, 6C2 (WBVR, Lelystad, Netherlands), was diluted 1:2000 in a commercial antibody diluent (Agilent-Dako, Santa Clara, CA) and applied to the slides for 15 min. Slides were developed with a BOND Polymer Refine Detection kit (Leica Biosystems, Buffalo Grove, IL) and counterstained with Mayer’s modified hematoxylin (Abcam, Cambridge, MA) diluted with water (1:4). Tissues were counterstained with hematoxylin.

### Fibril stability determination

Determination of the PrP^Sc^ fibril stability was conducted using a commercially available enzyme immunoassay/EIA kit (HerdChek®, IDEXX Laboratories Inc., Westbrook, ME) as previously described [52]. Three mice were selected for analysis from each treatment group based on the proximity of their survival time to the group mean incubation period. Whole brain homogenates were normalized to an EIA absorbance in the range of 0.8 to 1.5 and incubated in guanidine hydrochloride (GdnHCl) at the indicated concentration for 1 h. The samples were then diluted to 0.25 M GdnHCl, and the level of PrP^Sc^ remaining at each concentration was determined using EIA. Each mouse sample was analyzed in triplicate to ensure repeatable measures. The fraction of remaining fibril was determined by normalizing the O.D. values to the 0.25 M GdnHCl treatment point. Fibril stability is reported as the average concentration of GdnHCl at which 50% of the PrP^Sc^ remains in the fibril form ([GdnHCl_1/2_]). Tukey’s multiple comparison test for ordinary one-way ANOVA was used to determine the significance (alpha = 0.05) of differences in the mean fibril unfolding [GdnHCl_1/2_] for each group (GraphPad Prism 7, GraphPad Software, San Diego, CA).

### Western blot

In order to characterize the molecular properties of each inoculum group, we performed western blots to separate the three glycosylation states of PrP^Sc^. Samples had previously been homogenized to 20% (w/v) in PBS. For digestion of proteinase K-sensitive PrP, 0–5 μL of PBS and 1.5 μL of PK (1 mg/mL, ThermoFisher Scientific, Waltham, MA) were added to 15–20 μL of sample homogenate to create a 21.5 μL reaction. Samples were incubated at 37° Celsius for 1 h with constant mixing at 800 rpm. After incubation, the total volume of the solution was increased to 100 μL PBS and PK digestion was stopped with 1.5 μL of Pefabloc® (100 mg/mL) (Sigma-Aldrich, St. Louis, MO). Acetone precipitation of proteins was performed to enhance western blot banding patterns. Acetone was chilled to − 20° Celsius. Then 400 μL was mixed with 100 μL of each digested sample and incubated at − 20° Celsius for 1.5 h. The precipitated samples were spun at 15,000 rcf for 10 min. The supernatant was decanted, and the pellet was allowed to dry for 5–10 min. The pellets were resuspended in 30 μL of 1x loading buffer with 1.5 μL of β-mercaptoethanol. Samples were boiled at 100° Celsius for 5 min. NuPage 12% Bis-Tris precast gels (ThermoFisher Scientific, Waltham, MA) were loaded with 0.25–4 mg tissue equivalents of brain material per well. Gels were run at 200 V for 45 min in 1x MOPS running buffer. Proteins were transferred to low fluorescence PVDF membrane in a 10% methanol transfer buffer for 45 min at a constant 25 V. The membrane was probed with primary anti-PrP^Sc^ antibody 6H4 (diluted to a final concentration of 0.09 μg/ml, ThermoFisher Scientific, Waltham, MA), and incubated overnight at 4° Celsius. The remaining steps in the procedure were similar to previously described methods [13]. For the secondary incubation, we used a biotinylated anti-mouse antibody for 1 h (diluted to a final concentration of 0.1 μg/ml; Biotinylated anti-mouse IgG, Amersham Biosciences, USA) followed by incubation with streptavidin conjugated to horseradish-peroxidase for 1 h (diluted to a final concentration of 0.1 μg/ml; Streptavidin horseradish-peroxidase conjugate, Amersham Biosciences, USA). Horseradish peroxidase substrate (Pierce ECL-Plus, ThermoFisher Scientific, Waltham, MA) was used to develop a detectable signal that was imaged with a G:BOX gel imaging system (G:BOX Chemi-XT4, Syngene, Frederick, MD). Western blot analyses were performed with Image Lab™ Version 6.0.1 for Mac (Bio-Rad Laboratories, Inc., Hercules, CA).

## Supplementary information

**Additional file 1 A-B.** Lesion profiles are grouped to assist visualization and comparisons. **A.** The vacuolation profiles of o-bTME_VV_ (solid cyan line), L-BSE (solid black line), and bTME (dotted blue line) are similar. **B.** C-BSE and o-bTME_AV_ have distinct lesion patterns compared to bTME, o-bTME_VV_, and L-BSE. **C-G.** The mean (± SEM) for each isolate is plotted as a bold black line with each individual mouse appearing in light grey. Medulla (G1), cerebellum (G2), midbrain (G3), hypothalamus (G4), thalamus (G5), hippocampus (G6), para terminal body (G7), and cerebral cortex (G8 and G9). White matter in the cerebellar peduncle (W1), lateral tegmentum (W2), and the internal capsule (W3).

**Additional file 2.** Full length western blot from Fig. 7a. Lane 1, marker; lane 2, C-BSE; lane 3, o-bTMEAV; lane 4, o-bTMEVV; lane 5, bTME; lane 6, L-BSE; lane 7, marker.

## Data Availability

The datasets used and/or analyzed during the current study are available from the corresponding author on reasonable request.

## Abbreviations

TME: Transmissible mink encephalopathy
bTME: Bovine adapted transmissible mink encephalopathy
o-bTME: Ovine passaged bovine transmissible mink encephalopathy
o-bTME_VV_: Ovine passaged (VRQ/VRQ) bovine transmissible mink encephalopathy
o-bTME_AV_: Ovine passaged (VRQ/ARQ) bovine transmissible mink encephalopathy
C-BSE: Classical bovine spongiform encephalopathy
L-BSE: Atypical bovine spongiform encephalopathy, L-type
TgBovXV: Transgenic mouse expressing wildtype bovine PrP^C^
TSE: Transmissible spongiform encephalopathy
PrP^Sc^: Scrapie/disease associated prion protein (misfolded)
PrP^C^: Cellular prion protein, native
CJD: Creutzfeldt-Jakob disease
EIA: Enzyme immunoassay
TE: Transmission efficiency
AR: Attack rate
IP: Incubation period
GdnHCl: Guanidine hydrochloride
kDa: Kilodalton
OD: Optical density

## References

[1] Jeffrey M, Gonzalez L (2007). Classical sheep transmissible spongiform encephalopathies: pathogenesis, pathological phenotypes and clinical disease. Neuropathol Appl Neurobiol.

[2] Gibbs CJ, Gajdusek DC, Asher DM, Alpers MP, Beck E, Daniel PM (1968). Creutzfeldt-Jakob disease (spongiform encephalopathy): transmission to the chimpanzee. Science.

[3] Wells GA, Scott AC, Johnson CT, Gunning RF, Hancock RD, Jeffrey M (1987). A novel progressive spongiform encephalopathy in cattle. Vet Rec.

[4] Goldmann W, Hunter N, Foster JD, Salbaum JM, Beyreuther K, Hope J (1990). Two alleles of a neural protein gene linked to scrapie in sheep. Proc Natl Acad Sci U S A.

[5] Marsh RF, Hadlow WJ (1992). Transmissible mink encephalopathy. Revue scientifique et technique (International Office of Epizootics).

[6] Wilesmith JW, Ryan JB, Atkinson MJ (1991). Bovine spongiform encephalopathy: epidemiological studies on the origin. Vet Rec.

[7] Bruce ME, Will RG, Ironside JW, McConnell I, Drummond D, Suttie A (1997). Transmissions to mice indicate that 'new variant' CJD is caused by the BSE agent. Nature.

[8] Hill AF, Desbruslais M, Joiner S, Sidle KC, Gowland I, Collinge J (1997). The same prion strain causes vCJD and BSE. Nature.

[9] Collinge J, Sidle KC, Meads J, Ironside J, Hill AF (1996). Molecular analysis of prion strain variation and the aetiology of 'new variant' CJD. Nature.

[10] Hartsough GR, Burger D (1965). Encephalopathy of mink: I. Epizootiologic and clinical observations. J Infect Dis.

[11] Marsh RF, Bessen RA, Lehmann S, Hartsough GR (1991). Epidemiological and experimental studies on a new incident of transmissible mink encephalopathy. J Gen Virol.

[12] Baron T, Bencsik A, Biacabe AG, Morignat E, Bessen RA (2007). Phenotypic similarity of transmissible mink encephalopathy in cattle and L-type bovine spongiform encephalopathy in a mouse model. Emerg Infect Dis.

[13] Cassmann ED, Moore SJ, Smith JD, Greenlee JJ (2019). Sheep are susceptible to the bovine adapted transmissible mink encephalopathy agent by intracranial inoculation and have evidence of infectivity in lymphoid tissues. Front Vet Sci.

[14] Simmons MM, Chaplin MJ, Konold T, Casalone C, Beck KE, Thorne L (2016). L-BSE experimentally transmitted to sheep presents as a unique disease phenotype. Vet Res.

[15] Matsuura Y, Iwamaru Y, Masujin K, Imamura M, Mohri S, Yokoyama T (2013). Distribution of abnormal prion protein in a sheep affected with L-type bovine spongiform encephalopathy. J Comp Pathol.

[16] Capobianco R, Casalone C, Suardi S, Mangieri M, Miccolo C, Limido L (2007). Conversion of the BASE prion strain into the BSE strain: the origin of BSE?. PLoS Pathog.

[17] Lloyd SE, Linehan JM, Desbruslais M, Joiner S, Buckell J, Brandner S (2004). Characterization of two distinct prion strains derived from bovine spongiform encephalopathy transmissions to inbred mice. J Gen Virol.

[18] Beringue V, Andreoletti O, Le Dur A, Essalmani R, Vilotte JL, Lacroux C (2007). A bovine prion acquires an epidemic bovine spongiform encephalopathy strain-like phenotype on interspecies transmission. J Neurosci.

[19] Okada H, Masujin K, Miyazawa K, Yokoyama T (2015). Acquired transmissibility of sheep-passaged L-type bovine spongiform encephalopathy prion to wild-type mice. Vet Res.

[20] Espinosa JC, Andreoletti O, Castilla J, Herva ME, Morales M, Alamillo E (2007). Sheep-passaged bovine spongiform encephalopathy agent exhibits altered pathobiological properties in bovine-PrP transgenic mice. J Virol.

[21] Gonzalez L, Jeffrey M, Dagleish MP, Goldmann W, Siso S, Eaton SL (2012). Susceptibility to scrapie and disease phenotype in sheep: cross-PRNP genotype experimental transmissions with natural sources. Vet Res.

[22] Bruce ME, McConnell I, Fraser H, Dickinson AG (1991). The disease characteristics of different strains of scrapie in Sinc congenic mouse lines: implications for the nature of the agent and host control of pathogenesis. J Gen Virol.

[23] Gonzalez L, Chianini F, Hunter N, Hamilton S, Gibbard L, Martin S (2015). Stability of murine scrapie strain 87V after passage in sheep and comparison with the CH1641 ovine strain. J Gen Virol.

[24] Fraser H, Dickinson AG (1973). Scrapie in mice. Agent-strain differences in the distribution and intensity of grey matter vacuolation. J Comp Pathol.

[25] Peretz D, Scott MR, Groth D, Williamson RA, Burton DR, Cohen FE (2001). Strain-specified relative conformational stability of the scrapie prion protein. Protein Sci.

[26] Lezmi S, Bencsik A, Baron T (2006). PET-blot analysis contributes to BSE strain recognition in C57Bl/6 mice. J Histochem Cytochem.

[27] Nonno R, Marin-Moreno A, Carlos Espinosa J, Fast C, Van Keulen L, Spiropoulos J (2020). Characterization of goat prions demonstrates geographical variation of scrapie strains in Europe and reveals the composite nature of prion strains. Sci Rep.

[28] Fraser H, Dickinson AG (1968). The sequential development of the brain lesion of scrapie in three strains of mice. J Comp Pathol.

[29] Plinston C, Hart P, Chong A, Hunter N, Foster J, Piccardo P (2011). Increased susceptibility of human-PrP transgenic mice to bovine spongiform encephalopathy infection following passage in sheep. J Virol.

[30] Marin-Moreno A, Huor A, Espinosa JC, Douet JY, Aguilar-Calvo P, Aron N (2020). Radical change in zoonotic abilities of atypical BSE prion strains as evidenced by crossing of sheep species barrier in transgenic mice. Emerg Infect Dis.

[31] Bartz JC, Marsh RF, McKenzie DI, Aiken JM (1998). The host range of chronic wasting disease is altered on passage in ferrets. Virology.

[32] Hunter N, Goldmann W, Foster JD, Cairns D, Smith G (1997). Natural scrapie and PrP genotype: case-control studies in British sheep. Vet Rec.

[33] Hunter N, Moore L, Hosie BD, Dingwall WS, Greig A (1997). Association between natural scrapie and PrP genotype in a flock of Suffolk sheep in Scotland. Vet Rec.

[34] Bossers A, Schreuder BE, Muileman IH, Belt PB, Smits MA (1996). PrP genotype contributes to determining survival times of sheep with natural scrapie. J Gen Virol.

[35] Fediaevsky A, Tongue SC, Noremark M, Calavas D, Ru G, Hopp P (2008). A descriptive study of the prevalence of atypical and classical scrapie in sheep in 20 European countries. BMC Vet Res.

[36] Goldmann W, Hunter N, Smith G, Foster J, Hope J (1994). PrP genotype and agent effects in scrapie: change in allelic interaction with different isolates of agent in sheep, a natural host of scrapie. J Gen Virol.

[37] Bruce ME (2003). TSE strain variation. Br Med Bull.

[38] Baron T, Vulin J, Biacabe AG, Lakhdar L, Verchere J, Torres JM (2011). Emergence of classical BSE strain properties during serial passages of H-BSE in wild-type mice. PLoS One.

[39] Bessen RA, Marsh RF (1992). Identification of two biologically distinct strains of transmissible mink encephalopathy in hamsters. J Gen Virol.

[40] Huor A, Espinosa JC, Vidal E, Cassard H, Douet JY, Lugan S (2019). The emergence of classical BSE from atypical/Nor98 scrapie. Proc Natl Acad Sci U S A.

[41] Torres JM, Andreoletti O, Lacroux C, Prieto I, Lorenzo P, Larska M (2011). Classical bovine spongiform encephalopathy by transmission of H-type prion in homologous prion protein context. Emerg Infect Dis.

[42] Barrio T, Filali H, Otero A, Sheleby-Elias J, Marin B, Vidal E (2020). Mixtures of prion substrains in natural scrapie cases revealed by ovinised murine models. Sci Rep.

[43] Bruce M, Chree A, McConnell I, Foster J, Pearson G, Fraser H (1994). Transmission of bovine spongiform encephalopathy and scrapie to mice: strain variation and the species barrier. Philos Trans R Soc Lond B Biol Sci.

[44] Moore J, Tatum T, Hwang S, Vrentas C, West Greenlee MH, Kong Q (2020). Novel strain of the chronic wasting disease agent isolated from experimentally inoculated elk with LL132 prion protein. Sci Rep.

[45] Beck KE, Sallis RE, Lockey R, Simmons MM, Spiropoulos J (2010). Ovine PrP genotype is linked with lesion profile and immunohistochemistry patterns after primary transmission of classical scrapie to wild-type mice. J Neuropathol Exp Neurol.

[46] Kong Q, Zheng M, Casalone C, Qing L, Huang S, Chakraborty B (2008). Evaluation of the human transmission risk of an atypical bovine spongiform encephalopathy prion strain. J Virol.

[47] Castilla J, Gonzalez-Romero D, Saa P, Morales R, De Castro J, Soto C (2008). Crossing the species barrier by PrP (Sc) replication in vitro generates unique infectious prions. Cell.

[48] Safar J, Wille H, Itri V, Groth D, Serban H, Torchia M (1998). Eight prion strains have PrP (Sc) molecules with different conformations. Nat Med.

[49] Legname G, Nguyen HO, Peretz D, Cohen FE, DeArmond SJ, Prusiner SB (2006). Continuum of prion protein structures enciphers a multitude of prion isolate-specified phenotypes. Proc Natl Acad Sci U S A.

[50] Bett C, Joshi-Barr S, Lucero M, Trejo M, Liberski P, Kelly JW (2012). Biochemical properties of highly neuroinvasive prion strains. PLoS Pathog.

[51] Solforosi L, Milani M, Mancini N, Clementi M, Burioni R (2013). A closer look at prion strains: characterization and important implications. Prion.

[52] Vrentas CE, Greenlee JJ, Tatum TL, Nicholson EM (2012). Relationships between PrP^Sc^ stability and incubation time for United States scrapie isolates in a natural host system. PLoS One.

[53] Moore SJ, Vrentas CE, Hwang S, Greenlee MHW, Nicholson EM, Greenlee JJ (2018). Pathologic and biochemical characterization of PrPSc from elk with PRNP polymorphisms at codon 132 after experimental infection with the chronic wasting disease agent. BMC Vet Res.

[54] Ayers JI, Schutt CR, Shikiya RA, Aguzzi A, Kincaid AE, Bartz JC (2011). The strain-encoded relationship between PrP replication, stability and processing in neurons is predictive of the incubation period of disease. PLoS Pathog.

[55] Beringue V, Herzog L, Reine F, Le Dur A, Casalone C, Vilotte JL (2008). Transmission of atypical bovine prions to mice transgenic for human prion protein. Emerg Infect Dis.

[56] Hamir AN, Kunkle RA, Miller JM, Bartz JC, Richt JA (2006). First and second cattle passage of transmissible mink encephalopathy by intracerebral inoculation. Vet Pathol.

[57] Biacabe AG, Morignat E, Vulin J, Calavas D, Baron TG (2008). Atypical bovine spongiform encephalopathies, France, 2001-2007. Emerg Infect Dis.

[58] Buschmann A, Pfaff E, Reifenberg K, Muller HM, Groschup MH (2000). Detection of cattle-derived BSE prions using transgenic mice overexpressing bovine PrP(C). Arch Virol Suppl.

[59] Groschup MH, Buschmann A (2008). Rodent models for prion diseases. Vet Res.

[60] Cassmann ED, Moore SJ, Smith JD, Greenlee JJ (2019). Sheep with the homozygous Lysine-171 prion protein genotype are resistant to classical Scrapie after experimental Oronasal inoculation. Vet Pathol.

[61] Thackray AM, Hopkins L, Spiropoulos J, Bujdoso R (2008). Molecular and transmission characteristics of primary-passaged ovine scrapie isolates in conventional and ovine PrP transgenic mice. J Virol.

[62] Thackray AM, Hopkins L, Lockey R, Spiropoulos J, Bujdoso R (2011). Emergence of multiple prion strains from single isolates of ovine scrapie. J Gen Virol.

