# Supplementary figures and images for: Bovine adapted transmissible mink encephalopathy is similar to L-BSE after passage through sheep with the VRQ/VRQ genotype but not VRQ/ARQ
Source: BMC Vet Res. 2020 Oct 8;16:383. doi: 10.1186/s12917-020-02611-0 (PMC7545885; doi:10.1186/s12917-020-02611-0)

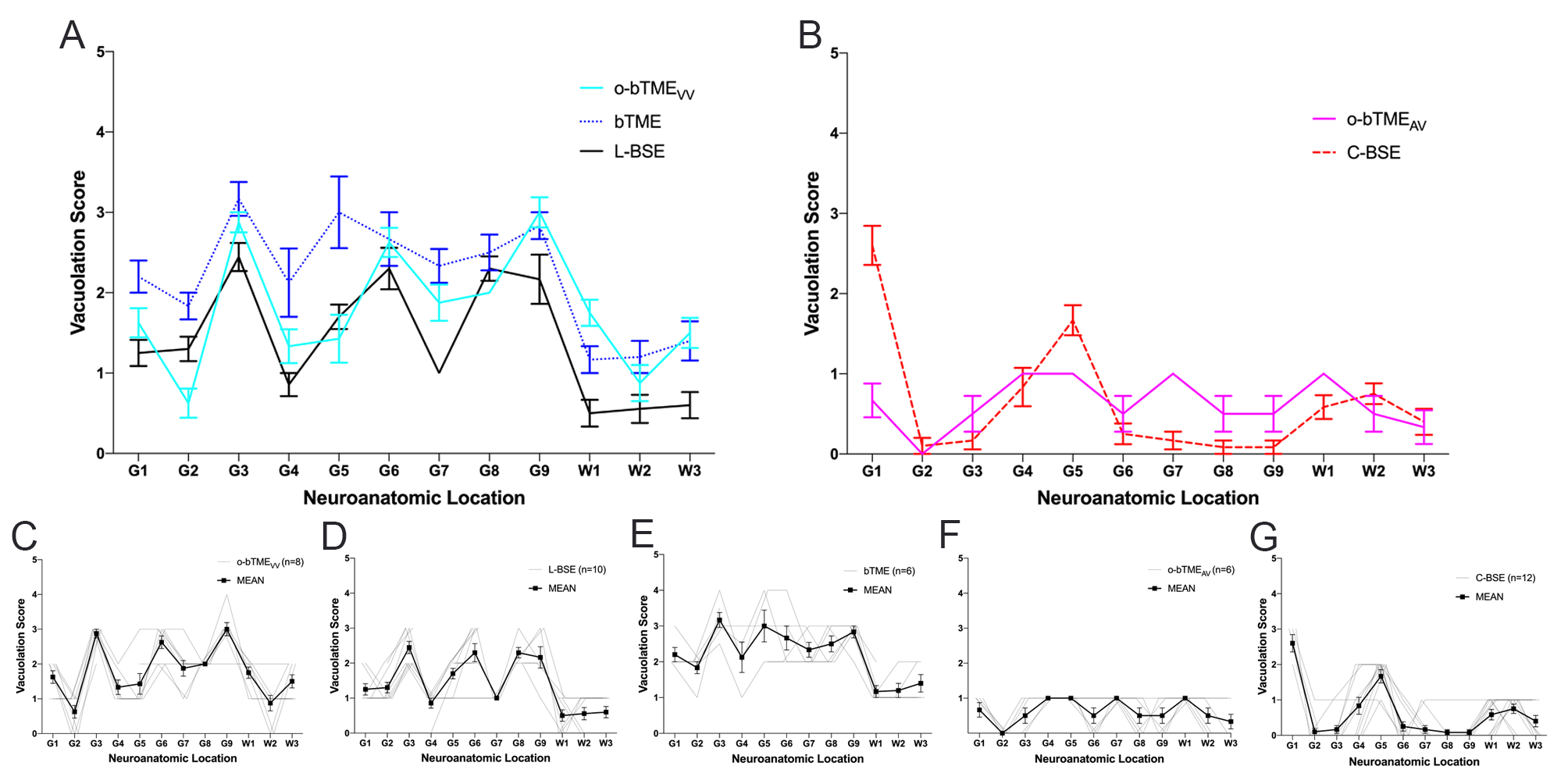

Supplement: Supplementary file 1 — Additional file 1 A-B. Lesion profiles are grouped to assist visualization and comparisons. A. The vacuolation profiles of o-bTMEVV (solid cyan line), L-BSE (solid black line), and bTME (dotted blue line) are similar. B. C-BSE and o-bTMEAV have distinct lesion patterns compared to bTME, o-bTMEVV, and L-BSE. C-G. The mean (± SEM) for each isolate is plotted as a bold black line with each individual mouse appearing in light grey. Medulla (G1), cerebellum (G2), midbrain (G3), hypothalamus (G4), thalamus (G5), hippocampus (G6), para terminal body (G7), and cerebral cortex (G8 and G9). White matter in the cerebellar peduncle (W1), lateral tegmentum (W2), and the internal capsule (W3). [file 12917_2020_2611_MOESM1_ESM.tif]

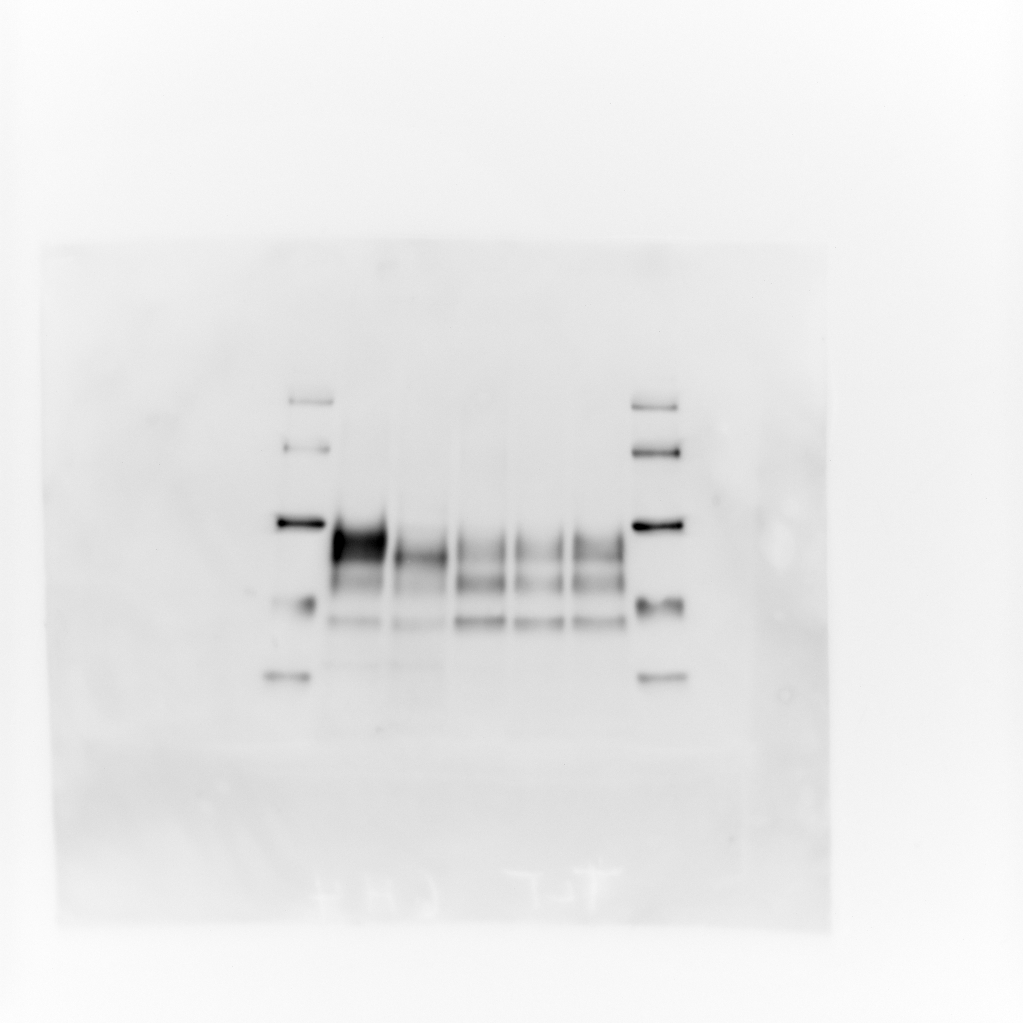

Supplement: Supplementary file 2 — Additional file 2. Full length western blot from Fig. 7a. Lane 1, marker; lane 2, C-BSE; lane 3, o-bTMEAV; lane 4, o-bTMEVV; lane 5, bTME; lane 6, L-BSE; lane 7, marker. [file 12917_2020_2611_MOESM2_ESM.tif]
